# Democratising data within a data-driven research centre: Examining the role of public engagement and involvement

**DOI:** 10.23889/ijpds.v9i5.2512

**Published:** 2024-09-18

**Authors:** Elizabeth Nelson

**Affiliations:** 1 Ulster University; 2 Administrative Data Research Centre Northern Ireland

Over the last decade there has been an increasing focus on utilising government-held data for large-scale data linkage research projects, with an emphasis on public benefit.

Most, if not all, administrative data research initiatives will recognise public involvement and engagement (PI&E) as a cornerstone of research and emphasise that administrative data is essentially public data, and, therefore, that publics must have a say in how it is used. Much of this focuses on engaging with communities and their representative organisations.

What is less explicitly discussed is the role that data-driven research plays within a broader public realm that is increasingly driven by data, or datafied.

This paper will examine the ways in which PI&E both challenges and contributes to the datafication of society, through the work of the Administrative Data Research Centre Northern Ireland (ADRC NI), part of ADR UK. It will explore if – and how  – PI&E in administrative data research can play a role in democratising this datafied society.

The paper will use the Northern Ireland Public Data Panel (NIPDP) and the Voices of Young People in Care (VOYPIC) initiative as case studies to explore the ways in which ADRC NI successfully or unsuccessfully create sites of democratisation within the data ecosystem.

Finally, the paper will consider how to amplify the democratic benefits of PI&E in data-driven research while minimising any potential harms, in the form of a potential framework for data justice within PI&E in data-driven research.

